# Process and outcome differences in the care of patients undergoing elective and emergency right hemicolectomy

**DOI:** 10.1308/rcsann.2024.0056

**Published:** 2024-07-31

**Authors:** J Banks, A Rashid, TR Wilson, CP Challand, MJ Lee

**Affiliations:** ^1^ Doncaster & Bassetlaw Teaching Hospitals NHS Foundation Trust, UK; ^2^ University of Sheffield, UK; ^3^ Sheffield Teaching Hospitals NHS Foundation Trust, UK

**Keywords:** Colon cancer, Outcomes

## Abstract

**Introduction:**

Up to 30% of patients with colorectal cancer present as an emergency and have worse outcomes than elective patients. Compared with left-sided cancers, malignancies arising in the right colon are significantly under-researched. We sought to compare cancer care quality and clinical outcomes between emergency and elective presentations of right-sided colon cancer (RCC).

**Methods:**

This multicentre, retrospective study included all patients who underwent operative management for a RCC, from 1 April 2017 to 31 March 2022. Data were collected from electronic patient records, and host and tumour factors as well as outcomes between emergency and elective cohorts were compared.

**Results:**

Overall, 806 patients (median age 72 years) were included. Some 175 patients (22%) presented as an emergency: 140 in obstruction and 35 with tumour perforation, compared with 1 patient with tumour perforation in the elective group (*p* < 0.001). The emergency group had higher rates of postoperative complications (59.1% vs 20.0%, *p* < 0.001), increased 90-day mortality (13.7% vs 1.3%, *p* < 0.001) and a longer hospital stay (5 vs 10 days, *p* < 0.001). From the emergency cohort only 29.2% of eligible patients received adjuvant chemotherapy and in multivariate regression analysis emergency presentation was associated with a decreased likelihood of receiving adjuvant chemotherapy (odds ratio 0.26 [0.14–0.47], *p* < 0.001).

**Conclusions:**

Both short- and long-term outcomes after emergency presentation of RCC are poor, with inadequate access to subsequent chemotherapy. Strategies addressing emergency presentations of left-sided tumours have moved towards temporisation and elective surgery. Delaying major resectional surgery for optimisation may improve outcomes and access to adjuvant therapies for RCC.

## Introduction

More than 1.1 million people are diagnosed with colon cancer (CC) globally each year, and it remains a leading cause of cancer deaths.^1^ Elective treatment pathways may include neoadjuvant therapy, surgery and adjuvant chemotherapy. In the United Kingdom (UK), the National Bowel Cancer Audit (NBOCA) reports that the percentage of deaths following treatment of colorectal cancer is 3.1%.^2^ Despite substantial efforts to improve early diagnosis and improve outcomes through bowel cancer screening programmes, 20% of patients in the UK still present as an emergency,^2^ including obstruction, perforation and haemorrhage.^2^ Emergency presentations are associated with poor perioperative outcomes and worse disease-specific and overall survival than presentations in the elective setting.^3^

In much published work on emergency colorectal cancer, studies pool data on all colorectal tumour sites. It is recognised that right and left CCs are different in nature.^4^ There is also evidence that right-sided colon cancers (RCCs) have worse prognosis than similarly staged left-sided CCs.^5^ This may reflect advanced stage, undetected microscopic disease or other factors related to treatment. There are currently limited data exploring the outcomes of right hemicolectomy for cancer in elective and emergency settings.

Given the specific challenges related to RCC, we intended to explore data from our own practice to identify areas where processes or outcomes differed, and opportunities to improve these. The aim of this study was to explore the surgical and adjuvant management of RCC.

## Methods

This study is reported with reference to the STROBE guidelines.^6^ As a service evaluation, ethical approval was not required, but local clinical effectiveness/audit approvals were secured (APPROVALS 1718/2022/SUR/KD).

### Patients

Data were obtained retrospectively for all patients who underwent operative management of a RCC, for both emergency and elective presentation, between April 2017 and March 2022 at two sites in the north of England. Both sites are large teaching hospitals with a total catchment >1.5 million patients. Data were collected from electronic patient records, potential cases were identified from hospital coding and the diagnosis of RCC was confirmed by corroborating with computed tomography (CT) scan results, operation notes and multidisciplinary team (MDT) documentation.

### Definitions

RCC was defined as an adenocarcinoma arising at a location from the caecum to the proximal transverse colon. Operative management included resection with a right hemicolectomy or extended right hemicolectomy (with or without stoma formation), defunctioning stoma with no resection and palliative bypass. Patients were divided into two groups, categorised by their presentation – emergency presentation with surgery being performed during the same admission, or elective presentation where MDT work-up was performed before the operation took place.

### Variables

Data were collected on patient factors (age, sex, ethnicity, body mass index, Charlston comorbidity score and American Society of Anesthesiologists' grade), tumour factors (American Joint Committee on Cancer stage, tumour location, tumour complications), cancer care pathway (preoperative CT imaging, colonoscopy, time from presentation to MDT discussion, time from MDT discussion to operation, number of high-risk patients referred for adjuvant chemotherapy, number of high-risk patients receiving chemotherapy) and operative factors (surgical approach, surgery specialty).

### Outcomes

Outcomes measured were mortality at 30 and 90 days post surgery, 3-year survival, postoperative complications as defined by treating clinical team and categorised by Clavien–Dindo classifications.^7^ Measured postoperative complications included infection (wound, chest or urinary), intra-abdominal collection, anastomotic leak, deep vein thrombosis/pulmonary embolism, ileus requiring total parenteral nuturiton, postoperative bleed and myocardial infarction. Length of hospital stay, and rates of adjuvant chemotherapy use with appropriateness referenced with National Institute for Health and Care Excellence (NICE) guidelines, were also measured.^8^

### Statistical analysis

Patient factors, tumour factors and the cancer care pathway were compared between emergency and elective groups to identify significant differences. Significance was set at *p* = 0.05 a priori. Continuous variables are presented as means or medians. Categorical variables are presented as absolute numbers (%). Comparative analysis of quantitative data was performed using Mann–Whitney *U* test and chi-squared test for proportions. Logistic regression analysis was performed to identify factors associated with the receipt of adjuvant chemotherapy between the two groups, controlling for patient age, comorbidities, cancer stage, surgical approach and postoperative complications. All data were analysed using R version 4.3.0.

## Results

Between April 2017 and March 2022, 806 patients underwent operative management for RCC, 175 patients (22%) presented as an emergency.

### Patient and tumour factors

The median age of patients was 72 (64–79) years and 384 (47.7%) of the patients were female. The comparison of patient factors between the two group is summarised in Table 1. Patients who presented as an emergency had more comorbidities; however, there was no difference between the two groups with regards to age, sex or ethnicity. Emergency presentation was associated with a more advanced tumour stage, and a higher likelihood of non-curative resection.

**Table 1:** Patient characteristics of 804 patients undergoing operative management of right-sided colon cancer

	Elective *n* = 631 (78%)	Emergency *n* = 175 (22%)	*p*-value
Age*	71 (65, 77)	73 (63, 80)	0.3
Sex			0.9
Female	301 (47.7)	83 (47.4)	
Male	330 (52.3)	92 (52.6)	
American Society of Anesthesiologists grade			0.5
1	125 (19.8)	28 (16.0)	
2	405 (64.2)	112 (64.0)	
3	90 (14.3)	31 (17.7)	
4	8 (1.3)	3 (1.7)	
Charlson Comorbidity Index*	5 (4, 7)	6 (5, 8)	**0**.**044**
Treatment intent			**0**.**004**
Curative	376 (59.6)	92 (52.6)	
Non-curative	255 (40.4)	83 (47.4)	
Cancer stage (AJCC)			**<0**.**001**
1	125 (19.8)	1 (0.6)	
2	245 (38.8)	52 (29.7)	
3	207 (32.8)	62 (35.4)	
4	54 (8.6)^b^	60 (34.3)^a^	

Values are given as n (%), except *n (). ^a^Age reported in years. Charlston Comorbidity Score 1–6. ^b^Age is reported in years. Charlston Comorbidity Score: 0, 1–2, 3–4 or >5.

### Cancer care pathway

Of the 806 included patients, 2 did not have a CT abdomen performed before their operation. Completion staging CT was performed significantly less frequently in the emergency cohort (92.6% vs 99.4%, *p* < 0.001). Preoperative colonoscopy was performed in 20.0% of emergency patients, which is expected given the emphasis on timely operative management in this cohort. Time from presentation to MDT discussion was shorter in the emergency cohort (15 days [10–21] vs 22 days [14–32], *p* < 0.001); however, 8 (6%) emergency patients were never discussed at MDT. The cancer care pathway comparison is summarised in Table 2.

**Table 2:** Comparison of the cancer care pathway for emergency and elective presentation

	Elective, *n* = 631 (78%)	Emergency, *n* = 175 (22%)	*p*-value
Diagnostic CT abdomen performed	629 (99.7)	175 (100)	1
Staging CT performed	627 (99.4)	162 (92.6)	**<0.001**
Preoperative colonoscopy	600 (95.1)	35 (20.0)	**<0.001**
Time from presentation to MDT discussion (days)*	22 (14, 32)	15 (10, 21)	**<0.001**
Time from presentation to operation (days)*	45 (25, 64)	1 (1, 3)	**<0.001**

Values are given as n (%), except *n (). CT = computed tomography; MDT = multidisciplinary team.

### Operative factors

Emergency presentation was associated with higher rates of open surgical resection and higher rates of stoma formation. The emergency cohort was significantly more likely to have a non-colorectal surgeon (upper gastrointestinal surgeon or emergency general surgeon) perform the operation (7.4% vs 0%, *p* < 0.001). The operative factors compared between elective and emergency presentation are summarised in Table 3.

**Table 3:** Operative factors for emergency and elective presentation

	Elective, *n* = 631	Emergency, *n* = 175	*p*-value
Operation performed			**<0.001**
Right hemicolectomy	572 (90.6)	94 (53.7)	
Right hemicolectomy with covering ileostomy or end ileostomy	50 (7.9)	52 (29.7)	
Defunctioning stoma, no resection	7 (1.1)	25 (14.3)	
Palliative bypass	1 (0.2)	4 (2.3)	
Operating surgeon speciality			**<0.001**
Colorectal	629 (99.7)	162 (92.6)	
Non-colorectal	0 (0)	13 (7.4)	
Surgical access			**<0.001**
Robotic	22 (3.5)	0 (0)	
Laparoscopic	459 (72.7)	53 (30.3)	
Laparoscopic converted to open	31 (4.9)	7 (4.0)	
Open	118 (18.7)	115 (65.7)	
Tumour perforation	9 (1.4)	37 (21.1)	**<0.001**

### Outcomes

**Figure 1:**
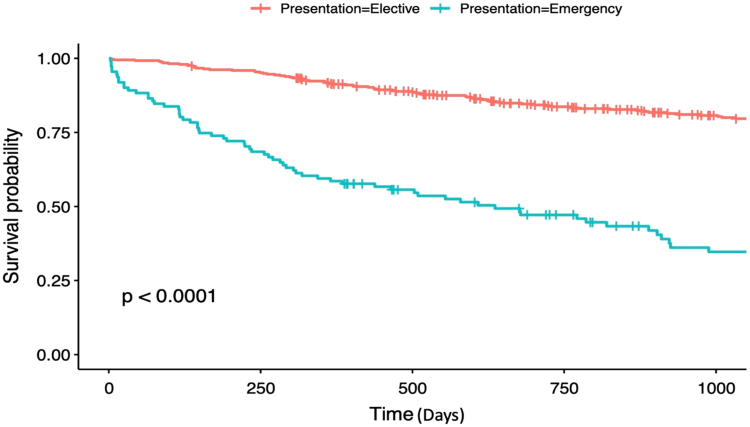
Kaplan–Meier survival plot of 3-year survival for elective and emergency presentation

Emergency presentation was associated with significantly higher postoperative complications (59.4% vs 20.0%, *p* < 0.001), and length of hospital stay (10 days [7–18] vs 5 days [4–8], *p* < 0.001) (Table 4). Postoperative mortality was significantly higher at both 30 and 90 days postoperatively in the emergency cohort (9.7% vs 1.0%, *p* < 0.001 and 13.7% vs 1.3%, *p* < 0.001, respectively). The significantly higher mortality in the emergency group extended beyond the immediate postoperative period; 3-year survival was significantly worse in the emergency cohort when compared with elective presentation (Figure 1).

**Table 4:** Summary of outcomes

Clinical outcomes	Elective, *n* = 631	Emergency, *n* = 175	*p*-value
Adjuvant chemotherapy rate (eligible patients as per NICE guidelines)	256/402 (63.7)	47/175 (29.2)	<0.001
Length of stay (days)*	5 (4, 8)	10 (7, 18)	<0.001
Postoperative complication	126 (20.0)	104 (59.4)	<0.001
30-day mortality	6 (1.0)	17 (9.7)	<0.001
90-day mortality	8 (1.3)	24 (13.7)	<0.001

Values are given as n (%), except *n (). NICE = National Institute for Heath and Care Excellence.

**Figure 2:**
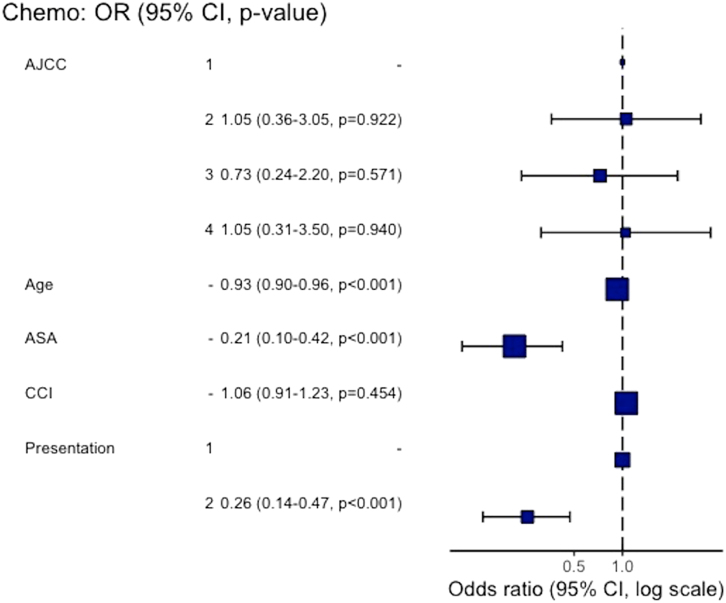
Multivariate logistic regression analysis of adjuvant chemotherapy receipt. For presentation 1 = elective and 2 = emergency. Odds ratio (OR) 0.93 (0.90 to 0.96), *p* < 0.001; OR 0.21 (0.10 to 0.42) *p* < 0.001; OR 0.26 (0.14 to 0.47) *p* < 0.001.

Rates of adjuvant chemotherapy were significantly lower in the emergency cohort (29.2% vs 63.7%, *p* < 0.001), with high rates of postoperative morbidity and mortality being the most common explanations cited at MDT for patients not being referred for chemotherapy. In multivariable regression, age (odds ratio [OR] 0.93 [0.90 to 0.96], *p* < 0.001)), American Society of Anesthesiologists grade (OR 0.21 [0.10 to 0.42], *p* < 0.001) and emergency presentation (OR 0.26 [0.14 to 0.47], *p* < 0.001) were associated with decreased likelihood of receiving adjuvant chemotherapy (Figure 2).

## Discussion

This study shows that emergency presentation of RCC is associated with significantly worse outcomes in both the immediate postoperative period and in the longer term. Given the frequency of this presentation, it is important to explore strategies to improve outcomes.

The rate of emergency presentation of RCC (22%) in our cohort is consistent with findings from previous studies, where emergency presentation ranges from 20% to 30%.^9–11^ Previous studies have identified that older patients, and patients with more comorbidities are more likely to present as an emergency.^12,13^ In our cohort there was no difference in age between emergency and elective presentation; however, the emergency cohort had a higher mean Charlson comorbidity score. Cancers presenting as an emergency were less likely to be stage 1, with a significant burden of stage 4 disease.

In terms of process, there were discrepancies between the two groups. Emergency presentations were less likely to be completely staged prior to surgery. In the emergency setting, the argument may be made that staging will not impact the immediate necessity for right hemicolectomy. However, knowledge of complete staging might influence decisions in the operative and perioperative phases. For example, knowledge of extensive pulmonary metastasis might prompt a surgeon to accept an R1 resection, where en bloc resection of adjacent viscera might otherwise be considered. Similarly, presence of metastatic disease might influence decisions on ceilings of care, including likelihood of surviving a reoperation for a major complication.^14^

This study was intended to explore the high-level question of variation in oncological pathways. Consequently, aspects of cancer biology are not fully addressed in this study. For example Lynch syndrome and major mismatch repair gene status are not presented here. There are also aspects of surgical technique and findings that have not been covered here; site of perforation or obstruction and its association with operative approach or outcomes, use of complete mesocolic excision vs standard resection approaches, and mitigating mesenteric injury with oedematous small bowel. Similarly, we do not have granular data on reasons for non-curative resections, but anticipate that many of these take place in the context of metastatic spread. It is not clear how presentation, biology and operative details will have influenced MDT decision making. All of these topics require further exploration in dedicated studies.

There was modest uptake of minimally invasive surgery in the emergency group in this study. Work from the NBOCA shows that around 30% of patients who underwent emergency surgery for colorectal cancer in 2016 had a laparoscopic approach.^15^ This was associated with shorter length of stay and reduced mortality. However, these benefits have yet to be demonstrated in a randomised controlled trial – the LaCeS trial is ongoing and may help resolve this argument.^16^ The limited use of laparoscopy may reflect service configuration, where a non-colorectal specialist may be asked to perform an emergency right hemicolectomy due to patient physiology demanding urgent action. Accordingly, this is a small group in this study, showing units' attempt to deliver specialist care associated with improved outcomes.^17^

Finally, the clinical outcomes noted for the emergency group should make surgeons pause in their decision making. More than half of all patients experienced an in-hospital complication, and mortality rates were approximately ten times higher than elective surgery at 30 and 90 days. As surgeons, we may be taught that a right hemicolectomy is a relatively simple resection, and therefore typically propose this as a primary approach. Lessons learned from the management of left-sided cancers, specifically temporisation with stent or stoma, potential neoadjuvant therapy, and definitive resection by a colorectal surgeon, do not seem to have translated to right-sided emergency presentations.^18^ In this data set, one in five emergency presentations had perforated. This is a group in which few options exist except resection. In those with tumours causing obstruction, perhaps other options might exist. Cohort data have shown the safety of an approach of stenting or defunctioning right-sided cancers.^19^ This temporisation may offer a chance to resolve acute physiological issues, reducing the risk profile of surgery. The recent FOXTROT trial has shown that neoadjuvant chemotherapy for colorectal cancer may lead to improved resectability of locally advanced disease, with a low risk of complications such as perforation or obstruction.^20^ Given the advanced state of disease in the emergency setting, it is conceivable that temporisation and neoadjuvant therapy may improve oncological outcomes in this subgroup.

### Study limitations

The study is not without limitations. The data are retrospective, and subject to the bias of those collecting initial data points in clinical notes. The study only provides outcomes from two high-volume centres. However, it provides an adequate sample size to inform reasonable estimates of clinical outcomes, and to identify process measures that might influence longer term clinical outcomes.

Policy makers and researchers should consider whether this is an area of priority for action, or whether to accept the status quo demonstrated here and elsewhere, and persistently so over the past 15+ years.^9,21^ Exploration of a policy of temporisation and neoadjuvant therapy where possible might provide some benefit. In the interim, strategies to optimise outcomes should be employed where possible.^18^

### Conclusion

Despite significant efforts to improve early detection of colorectal cancer, a significant proportion presents as an emergency. Emergency presentation of right-sided colorectal cancer is significantly under-researched compared with the left side. This article demonstrates the persisting gap in outcomes between emergency and elective presentations of RCC and highlights the need to consider a new treatment approach with this presentation.
